# Lack of Detectable HIV-1 Molecular Evolution during Suppressive Antiretroviral Therapy

**DOI:** 10.1371/journal.ppat.1004010

**Published:** 2014-03-20

**Authors:** Mary F. Kearney, Jonathan Spindler, Wei Shao, Sloane Yu, Elizabeth M. Anderson, Angeline O'Shea, Catherine Rehm, Carry Poethke, Nicholas Kovacs, John W. Mellors, John M. Coffin, Frank Maldarelli

**Affiliations:** 1 HIV-1 Drug Resistance Program, Center for Cancer Research, National Cancer Institute, Frederick, Maryland, United States of America; 2 Advanced Biomedical Computing Center, SAIC, Frederick, Maryland, United States of America; 3 Laboratory of Immunoregulation, National Institute of Allergy and Infectious Diseases, NIH Bethesda, Maryland, United States of America; 4 Department of Medicine, University of Pittsburgh, Pittsburgh, Pennsylvania, United States of America; 5 Department of Molecular Biology and Microbiology, Tufts University, Boston, Massachusetts, United States of America; John Hopkins University, United States of America

## Abstract

A better understanding of changes in HIV-1 population genetics with combination antiretroviral therapy (cART) is critical for designing eradication strategies. We therefore analyzed HIV-1 genetic variation and divergence in patients' plasma before cART, during suppression on cART, and after viral rebound. Single-genome sequences of plasma HIV-1 RNA were obtained from HIV-1 infected patients prior to cART (N = 14), during suppression on cART (N = 14) and/or after viral rebound following interruption of cART (N = 5). Intra-patient population diversity was measured by average pairwise difference (APD). Population structure was assessed by phylogenetic analyses and a test for panmixia. Measurements of intra-population diversity revealed no significant loss of overall genetic variation in patients treated for up to 15 years with cART. A test for panmixia, however, showed significant changes in population structure in 2/10 patients after short-term cART (<1 year) and in 7/10 patients after long-term cART (1–15 years). The changes consisted of diverse sets of viral variants prior to cART shifting to populations containing one or more genetically uniform subpopulations during cART. Despite these significant changes in population structure, rebound virus after long-term cART had little divergence from pretherapy virus, implicating long-lived cells infected before cART as the source for rebound virus. The appearance of genetically uniform virus populations and the lack of divergence after prolonged cART and cART interruption provide strong evidence that HIV-1 persists in long-lived cells infected before cART was initiated, that some of these infected cells may be capable of proliferation, and that on-going cycles of viral replication are not evident.

## Author Summary

Anti-HIV compounds are highly effective for preventing the onset of AIDS but they do not cure infected individuals. Very low levels of virus remain detectable in the blood of most patients despite antiviral treatment and levels surge if treatment is stopped. It is crucial to understand why current treatments are not equipped to cure HIV infection so that new therapies addressing these shortcomings can be developed. By characterizing genetic sequences of HIV in patients before and during antiviral treatment, we found that the low levels of virus detected in the blood of treated patients did not result from newly infected cells but originated from cells, or the daughters of cells, that were already infected when treatment was initiated. This finding demonstrates that HIV present in blood after prolonged antiviral treatment is derived from cells infected prior to treatment which likely expanded over time through cell division. Such long lived, infected cells are likely the critical target for developing strategies to cure HIV infection.

## Introduction

The HIV-1 lifecycle includes rapid and error prone nucleic acid replication that results in large and genetically diverse virus populations *in vivo*. The consequences of broad HIV-1 genetic diversity include the presence of viral variants containing mutations that escape immune responses or confer resistance to individual antiretroviral agents. The use of antiretroviral agents in combination results in potent suppression of HIV-1 replication and reverses immune deficiency, at least in part. Despite the ability of cART to inhibit HIV-1 replication, treatment does not eradicate infection and plasma viremia persists at low levels in the majority of patients [1], [2]. If cART is discontinued, viremia rapidly rebounds to pre-therapy levels [3], [4]. Determining the sources and mechanisms for viral persistence during cART and rebound after interruption is essential for designing strategies to eradicate infection.

The dynamics of HIV-1 decay after initiating cART can be divided into four phases [1], [2], [5]. The first phase, reflecting rapid clearance of ca 90% of productively infected cells with half-life of 1–2 days, is followed by a more gradual clearance of infected cells with a half-life of 2–3 weeks. A study by Palmer, *et al.* described a third phase consisting of long-lived, perhaps latently-infected, cells with a half-life of 6–44 months as well as a fourth phase having a slope not significantly different from zero [1]. The plateau in the fourth phase suggests that long-term cART fully inhibits HIV-1 replication and that the source of persistent viremia is either long-lived virus-expressing cells or activation of virus expression from latently-infected cells. In this regard, studies by Dinoso *et al.*, McMahon *et al.*, and Gandhi *et al.* showed no decrease in the level of persistent viremia in patients on long term suppressive therapy before, during, or after intensification with an additional antiretroviral suggesting the absence of ongoing new rounds of replication during suppressive cART [6], [7], [8]. Bailey *et al.* investigated plasma viral sequences after long-term cART and found that HIV-1 populations often contain sets of identical sequences, referred to as “predominant plasma clones,” suggesting that viral subpopulations are lost over the course of treatment [9]. Wagner, *et al*. found an increasing frequency of identical sequences in blood cells during cART suggesting proliferation of infected cells [10], and Joos, *et al.* showed that homogeneous populations rebound after cART interruption [11]. These findings suggest that a reservoir of long lived infected cells, perhaps capable of expansion, may be responsible for persistent viremia and its rebound following interruption of cART.

In contrast to these findings, other studies have indicated that low-level virus replication may occur in specific anatomical compartments despite suppression of plasma HIV-1 RNA by cART [12], [13], [14], [15], [16], [17], [18], [19], [20]. For example, in 2008, Chun, *et al.* suggested that phylogenetic clustering of sequences obtained from different cellular compartments after long-term cART demonstrated cross-infection between reservoirs, consistent with full cycles of replication as a source of persistent viremia [13]. Although such phylogenetic clustering may be indicative of on-going replication, it may also result from compartmental mixing of infected cells before or subsequent to initiating therapy. Demonstrating the emergence of new viral variants during cART without corresponding increases in total HIV-1 RNA would provide clear evidence of virus replication. Previous studies that demonstrated genetic change during therapy were in the context of drug resistance, rebound viremia, or stimulation following vaccination, each occurring in subsets of study patients in conjunction with increases in plasma HIV-1 RNA levels, likely reflecting ineffective therapy [12], [14], [21], [22]. Several studies using integrase inhibitors to intensify cART have detected transient increases in 2-LTR circles in peripheral blood lymphocytes, especially in individuals undergoing protease inhibitor-based cART suggesting that some cells may be newly infected during treatment [23] [24]. However, changes in 2LTR circles were not associated with decreases in viral RNA levels and genetic analyses did not show divergence during the intensification period [25]. Notably, all of these clinical studies have been conducted with patients already undergoing cART for prolonged periods. No studies have investigated HIV-1 populations prior to and following initiation of cART. Comparing pre- and post-therapy populations can shed new light on HIV-1 reservoirs, the sources of persistent viremia, and changes in HIV-1 populations at each phase of viral decay after introducing cART.

To investigate further the effect of cART on virus replication, we examined HIV-1 populations in patients prior to cART, during each phase of viral decay including long-term cART (fourth phase), and during viral rebound after interruption of cART. By investigating the genetics of HIV-1 in all phases of viral decay and comparing on-therapy populations to pre-therapy virus we were able to directly assess HIV-1 replication and molecular evolution during long-term suppressive cART. We found that both short and long lived cellular compartments were seeded with the same diverse virus populations and that new viral populations rarely emerged after up to 15 years of cART.

## Materials and Methods

### Study participants

Participants were enrolled in prospective studies aimed at determining the role of antiretroviral therapy on HIV-1 infection (protocols 97-I-0082, 08-I-0221) or on HIV-1 population genetics in infected individuals (00-I-0110) conducted at the NIH Clinical Center in Bethesda MD [26] [27]. All participants were ≥18 years of age at study entry, with chronic HIV-1 infection (Fiebig Stage VI) and reported no prior antiretroviral therapy (Table 1). Study participants were enrolled from 1997–2002; Patients 2–4 and 6–13 initiated therapy with 2 NRTIs + nevirapine + indinavir as part of a study of HIV-1 decay kinetics [26] and Patients 1, 5, and 14 initiated therapy with 2 NRTIs + efavirenz as part of a study of HIV-1 population genetics [27] (Table 1). Frequent plasma samples were obtained prior to and following introduction of cART (Supplemental Table S1). Patients are described in Table 1 and samples analyzed in Supplemental Table S1. Patients were categorized into three partially overlapping groups according to their sample collection and treatment history (Table S1). Blood samples were collected prior to initiating cART in all patients (N = 14). In 10/14 patients (group 1) frequent samples were collected during short-term treatment (up to one year on cART). In 5 patients (group 2), samples were collected after long-term therapy (average 9 yrs on cART), and in 5 patients (group 3), samples were collected after a patient-initiated treatment interruption as well as after re-suppression in 3/5 (Table S1). Results from the sequence analysis from all groups were compared to data obtained using the same methods from a cohort of elite controllers (data previously published) [28]. The elite controllers served as untreated controls since they have similar levels of viremia (mean 0.8 copies/ml) without cART.

**Table 1:** Characteristics of study participants.

PID	Age	Gender	Race	Ethnicity	Risk	Baseline HIV RNA (Log10 copies/ml)	Baseline CD4 (cells/µl)	CD4 (before <50 HIV RNA c/mL, in cells/µl)
**1**	41.2	Male	Black	Not Hispanic or Latino	MSM	5.2	64	167
**2**	51.5	Male	Black	Not Hispanic or Latino	MSM	5.6	23	89
**3**	36.2	Male	Unknown	Hispanic or Latino	MSM	4.7	607	617
**4**	28.5	Male	Black	Not Hispanic or Latino	Heterosexual	4.4	440	649
**5**	40.2	Male	Black	Not Hispanic or Latino	MSM	6.3	101	277
**6**	43.5	Male	Black	Not Hispanic or Latino	Heterosexual	5.2	11	93
**7**	38.2	Female	White	Not Hispanic or Latino	Heterosexual	5.7	6	135
**8**	48.3	Male	White	Not Hispanic or Latino	MSM	3.7	418	596
**9**	33.2	Male	White	Not Hispanic or Latino	MSM	4.8	202	375
**10**	44.6	Male	White	Not Hispanic or Latino	MSM	4.8	242	517
**11**	46.5	Male	Black	Not Hispanic or Latino	MSM	5.5	18	32
**12**	40.2	Male	White	Not Hispanic or Latino	MSM	4.1	616	808
**13**	36.9	Male	White	Not Hispanic or Latino	MSM	4.1	784	671
**14**	30.6	Male	Unknown	Hispanic or Latino	Bisexual	5.8	137	218
**Median**	**40.2**					**5.0**	**169.5**	**326.0**

### Ethics statement

All participants in this study were enrolled in clinical protocols (00-I-0110, 97-I-0082, 08-I-0221) approved by the NIAID Institutional Review Board (FWA00005897) administered at the NIH Clinical Center in Bethesda, Maryland. Individuals underwent an informed consent process and provided written consent for participation.

### HIV-1 genetic analyses of plasma samples

HIV-1 RNA levels were determined using bDNA Versant version 3.0 (Bayer, Inc) as previously described [29]. Single-genome sequencing (SGS) of a portion of HIV-1 *gag-pro-pol* amplified from plasma HIV-1 RNA was performed as previously described [30], [31], [32]. Sequences were aligned using ClustalW. Population genetic diversity and divergence were calculated as average pairwise difference (APD) using MEGA5 [33] (http://www.megasoftware.net) and an in-house program [32]. Shifts in population structure were calculated using a subdivision test for panmixia with a significance cut off level of p<10^−3^ as described by the original report to account for the high number of comparisons between sequences and nucleotide sites [34], [35] [27]. The probability of 10^−3^ for assigning a significant change in intra-patient HIV populations was derived statistically taking into consideration that every nucleotide position is compared in every two possible sets of sequences. This approach results in more than 10^12^ comparisons between populations of only 10 sequences. The test was derived from a geographic population structure test proposed by Hudson *et al.* [36]. It compares the APD in single-genome sequences obtained from samples taken at different times (or places) to distances calculated from imaginary populations containing the same sequences randomly reassigned to two groups. Random mixing of the populations to be compared, reassignment, and distance comparisons are performed 10,000 times, generating a *p*-value for the probability that the randomized populations' structures are the same between sets of sequences. Neighbor-joining phylogenetic analyses were performed using MEGA5 [33]. Trees were rooted on the subtype B consensus sequence (http://www.HIV-1.lanl.gov). Tests for molecular evolution were done with BEAST [37] (http://beast.bio.ed.ac.uk) using the HYK+G model with a relaxed clock, uncorrelated log normal and constant size, followed by estimating the root to tip distances with TreeStat1.2 (www.tree.bio.ed.ac.uk/software/treestat). Linear regression was used to determine the slopes for the root-to-tip analyses.

To investigate if genetic bottlenecks occurred after initiating cART, we evaluated changes in the number of heterozygous sites over time [38]. A genetic bottleneck was present if the number of heterozygous sites in equal numbers of sequences in post-therapy samples were in excess (chi-square probability <0.05) compared to those in pre-therapy. We also investigated if CTL escape mutations were enriched or depleted during cART by calculating the allele frequencies at each amino acid position in pre-therapy and post-therapy data in patients in group 1 and 2 with 7 or more sequences at distal time points (N = 8). Positions with amino acids undergoing significant change in frequency after cART (Fisher exact test 0.05) were identified and mapped onto predicted CTL epitope maps [39]. Changes within the 9 amino acid peptide or in +1 and -1 amino acids flanking the peptide were considered to be part of the CTL epitope. The predicted binding affinities of the pre- and post-therapy peptides were compared to determine if amino acid changes occurring after initiating cART resulted in decreased binding affinity; ≥10 fold decreases in affinity were considered escape; ≥10 fold increases were considered return to wild-type allele.

## Results

### Effect of cART on plasma HIV-1 diversity

**Figure 1:**
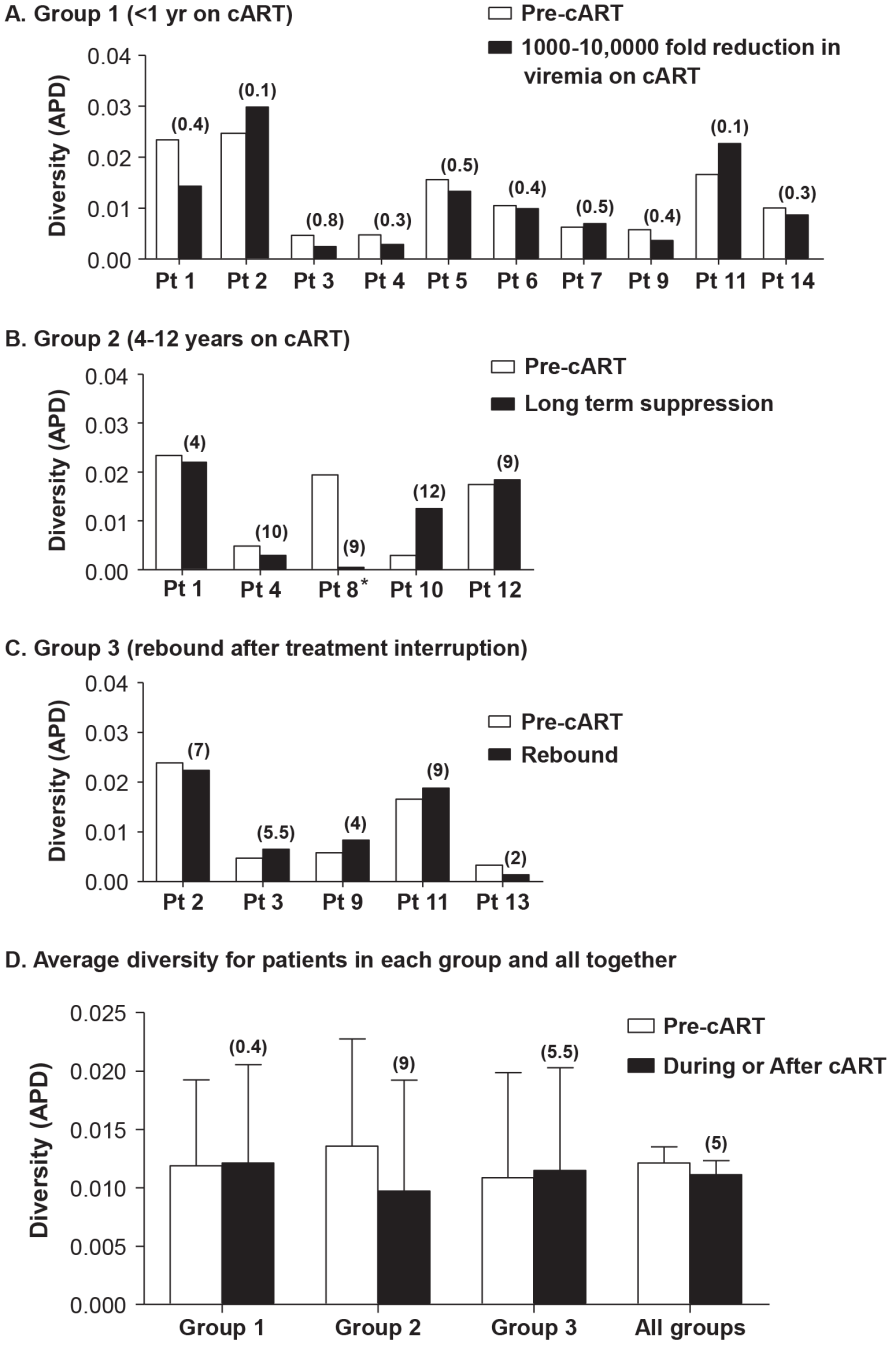
Measurements of HIV-1 diversity calculated as APD before, during and/or after cART in all patients in (A) Group 1 - short-term cART (B) Group 2 - long-term cART (C) Group 3 - cART with treatment interruptions and (D) the average of all groups. Duration of treatment is shown in years in parentheses above the bar with the diversity measurement. Overall, HIV-1 plasma diversity did not change with initiation of therapy.

**Figure 2:**
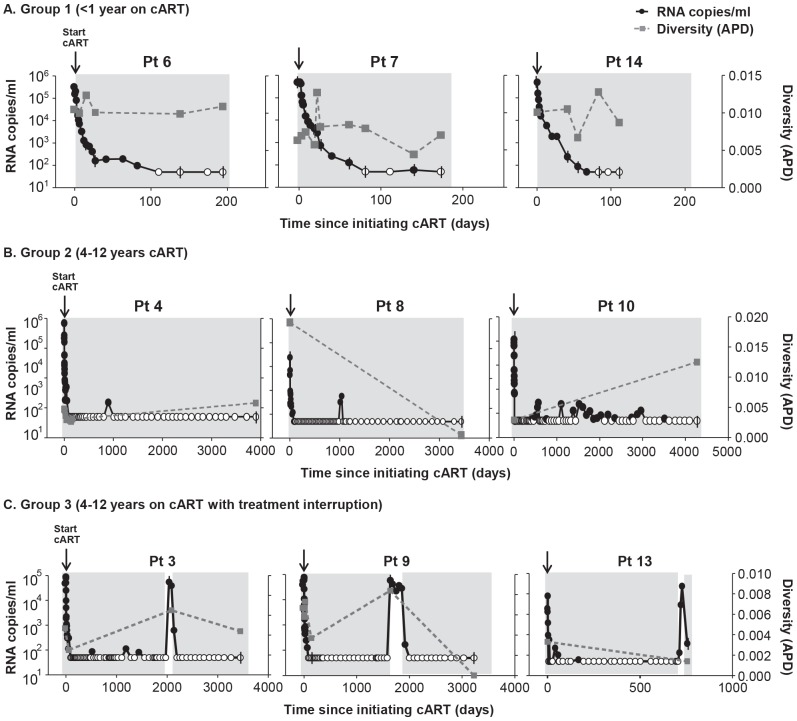
HIV-1 plasma RNA copy numbers and diversity as calculated by APD in longitudinal samples prior to and during cART in selected patients on (A) short-term cART (Group 1) (B) long-term cART (Group 2) and (C) cART with treatment interruptions (Group 3). We found no relationship between HIV-1 RNA copy number and viral diversity in the plasma.

To investigate the effect of cART on plasma HIV-1 diversity, we assessed HIV-1 genetics by single-genome sequencing of plasma HIV-1 RNA in individuals undergoing cART. Plasma samples were obtained prior to and following introduction of cART; and, for some patients, after planned patient-initiated treatment interruptions. Single-genome sequences were obtained at time points throughout the study period, and population genetics parameters were measured. Genetic diversity was measured by APD of virus populations in patients' plasma prior to treatment, during each phase of viral decay, and during viral rebound (Figures 1, 2). Group 1 patients were sampled during the first and second phases of HIV-1 decay on cART (up to 200 days) to investigate the effect of declining viremia on virus diversity (Figure 1a, 2a). Group 2 patients were sampled on long term cART (c. 4–12 years) without treatment interruption during the third and/or fourth phases of viral decay (Figure 1b, 2b). Group 3 patients with long-term suppression of HIV-1 underwent brief planned treatment interruptions and were sampled before and after treatment initiation and after virus rebound (Figure 1c, 2c).

Most patients (13 of 14) showed no significant difference in APD of HIV-1 populations during any phase of viral decay, after long-term therapy, or after viral rebound, compared to pre-therapy virus populations (Figure 1, 2). This finding shows, in most cases, that HIV-1 plasma diversity is not associated with the level of viremia (Figure 2), with the duration of cART, or with viral rebound after stopping cART. Figure 1 shows the diversity of plasma HIV-1 populations in each patient before and during or after interruption of cART (the value above the bar in Figure 1 shows the number of years the sample was collected after initiating cART). Of 14 patients, only one (PID 8) showed a significant reduction in viral diversity after treatment with cART (Figure 1b, 2b). The mean virus diversity across patients in each group and as a whole did not change after initiation of cART or during cART (Figure 1d), indicating that plasma virus diversity is sustained during each phase of viral decay despite the large decreases in the replicating population size. This result suggests that the cellular reservoir of persistent viremia in most patients is seeded with the same highly diverse replicating population of virus that exists prior to therapy. This observation is in contrast to elite controllers who have significantly lower levels of diversity than noncontrollers (p = 0.005) [28] correlating with their lower levels of viremia. The contrasting results suggest that the infected cell population in patients treated with cART is large while the reservoir of infected cells in elite controllers is likely to be significantly smaller.

### Effect of cART on number of alleles

Although HIV-1 populations revealed no significant change in APD with cART, genetic bottlenecks may occur in large, diverse populations without producing a detectable change in the overall diversity. During a bottleneck, low frequency alleles, which do not contribute substantially to overall diversity or to phylogenetic signal, are lost [38]. As a result, the total number of alleles is decreased while the diversity is maintained. Because bottlenecks will have substantial effects on the occurrence of low frequency alleles, we specifically investigated the total number of alleles prior to and following introduction of cART (Table 2) in patients with sampling during viral RNA decay on therapy (N = 9). We found a significant decrease in the numbers of alleles in only a single patient (PID 1), suggesting that a genetic bottleneck occurred in this patient alone. In two patients (PID 2 and 7), a modest but detectable increase in alleles occurred suggesting genetic shifts but not population contraction. The remaining patients had no changes in the numbers of alleles (Table 2), indicating that, for the majority of individuals, no genetic bottleneck accompanies the profound decrease in HIV RNA after initiation of cART.

**Table 2:** Allele frequency during cART.

Patient^a^	Therapy Period	Number of Sequences	Total Number of Alleles	Total Number of Homozygous Sites	Heterozygosity	Chitest
1	Pre therapy	38	546	978	0.13	
1	Post therapy	38	415	986	0.13	0.0004
2	Pre therapy	12	146	1010	0.10	
2	Post therapy	12	197	987	0.12	0.01
4	Pre therapy	49	124	1021	0.08	
4	Post therapy	49	105	1062	0.05	0.14
5	Pre therapy	99	1077	885	0.21	
5	Post therapy	99	1109	836	0.25	0.18
6	Pre therapy	45	323	1010	0.10	
6	Post therapy	45	283	1009	0.10	0.16
7	Pre therapy	23	87	1056	0.06	
7	Post therapy	23	117	1037	0.08	0.03
9	Pre therapy	31	128	1036	0.08	
9	Post therapy	31	109	1021	0.09	0.29
11	Pre therapy	22	248	1016	0.10	
11	Post therapy	22	262	1011	0.10	0.55
14	Pre therapy	20	130	1035	0.08	
14	Post therapy	20	129	1038	0.08	0.94

a Analysis includes only patients who had sampling during viral RNA decay on cART.

To specifically investigate whether prolonged HIV-1 suppression resulted in changes in amino acid sequences, we investigated nonsynonymous changes alone in patients from groups 1 and 2 for which there were more than 7 sequences at time points with <50 copies/ml (N = 8) (Table S2). We found that amino acid frequencies were remarkably stable during cART. In fact, virus populations in 4/8 patients had no significant change at any of the PR or RT loci. As all enrolled patients underwent HLA testing, we were able to investigate, using in silico techniques, the predicted positions of all the CTL epitopes in the HIV-1 sequence as well as the estimated binding affinity of all the HIV-1 peptides at each epitope site [39]. As shown in Table S2, there was no consistent trend to enrich or deplete CTL escape mutations after prolonged cART suppression, including in those patients who underwent a significant population shift (e.g., PID 1). Taken together, these data suggest that the population of virus-producing cells present after prolonged suppression is not shaped in a substantial way by new CTL selection following introduction of cART. This finding is in stark contrast to the strong selection at CTL epitopes in elite controllers ranging from 11–66% of epitopes carrying escape mutations [28].

### Effect of cART on HIV-1 population structure and divergence

**Figure 3:**
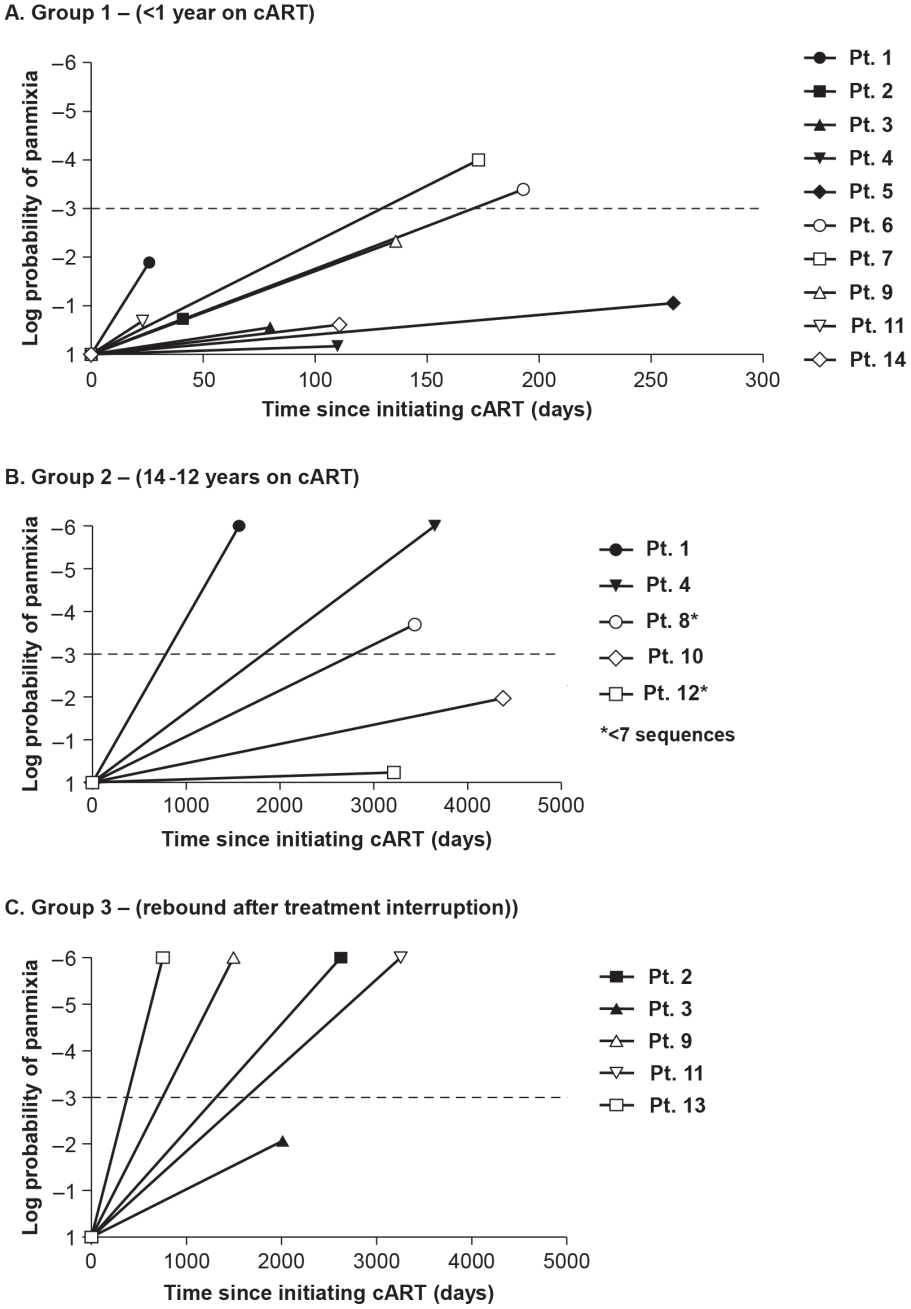
Measurements of the probability of panmixia before and after cART in (A) Group 1 - short-term cART (B) Group 2 - long-term cART (C) Group 3 - cART with treatment interruptions. We considered a panmixia p value of <0.001 to be statistically significant according to the original publication of the method (1). Significance reveals a shift in population over time on cART and was found primarily in patients on long-term cART.

Divergence of HIV-1 populations during cART could result either from on-going cycles of replication leading to the emergence of new variants or as a consequence of shifts in the viral variants present in the plasma during suppression, indicating a dynamic reservoir. To investigate the possibility of population shift (divergence) during cART, we used a test for panmixia to detect changes in the population structure during therapy compared to pretherapy virus. The panmixia test compares populations of single-genome sequences obtained from longitudinal samples and provides a *p*-value for the probability that the populations are the same [34]. Probabilities of <10^−3^ were considered to indicate significantly different populations, taking into account the large numbers of comparisons. Figure 3 and Table 3 show the panmixia results for single-genome sequences from group 1 (Figure 3a, Table 3), group 2, (Figure 3b, Table 3), and group 3 (Figure 3c, Table 3) compared to pretherapy sequences. Panmixia probabilities of virus populations in samples collected from patients on cART compared to pre-therapy populations did not achieve significance (Figure 3a) in 8/10 patients from group 1. These results indicate that there is typically no significant shift in the plasma virus population during the first and second phases of decay after initiating cART despite up to 10,000-fold declines in levels of viremia. Two patients in group 1 (PID 6, 7), however, did show a significant change in population structure after 173 and 193 days on therapy. Additional analyses describing the nature of these changes are presented below. Three of 5 patients in group 2 (long-term cART) showed a significant change in population structure during cART for 4–12 years with no treatment interruptions, suggesting either that new variants emerged during therapy or that the reservoir for persistent viremia is dynamic. Four of 5 patients in group 3 (long-term cART but with brief treatment interruptions) showed a significant shift in population structure using the panmixia test. The results from group 2 and 3 show that, although plasma HIV-1 populations do not typically change in the early phases of viral decay, shifts in virus populations (without a change in overall diversity) are readily detectable after long-term therapy and in rebound viremia. They imply that either a compartment allowing on-going cycles of replication exists during cART or subsets of infected cells expressing virus particles shift over the course of treatment (through proliferation and/or death).

**Table 3:** Divergence from pretherapy virus.

PID	Group^a^	Sample Description	Probablility of Panmixia	Root to Tip Slope^d^
1	1	26 days on cART	0.0134	0.2
2	1	41 days on cART	0.4505	−0.03
3	1	80 days on cART	0.0609	−0.0006
4	1	110 days on cART	0.4032	−0.001
5	1	260 days on cART	0.1358	0.002
6	1	193 days on cART	0.0006*	0.0001
7	1	173 days on cART	0.0003*	−0.0009
9	1	136 days on cART	0.258	−0.0002
11	1	23 days on cART	0.4927	−0.02
14	1	111 days on cART	0.0733	−0.0007
1	2	1566 days on cART	**<0.000001**	0.02
4	2	3650 days on cART	**<0.000001**	0.00009
8	2	3437 days on cART	**<0.000001** b	0.00008
10	2	4380 days on cART	0.3311	0.00003
12	2	3215 days on cART	0.573^c^	−0.002
2	3	Rebound viremia after 2624 days on cART	**<0.000001**	0.005
3	3	Rebound viremia after 2013 days on cART	0.0085	0.0009
9	3	Rebound viremia after 1497 days on cART	**<0.000001**	0.001
11	3	Rebound viremia after 3255 days on cART	**0.00001**	0.002
13	3	Rebound viremia after 755 days on cARY	**<0.000001**	0.002

a Group definitions: Group 1 - Samples analyzed pre-cART and <1 yr on cART. Group 2 - Pre-cART and after long-term suppression on cART (average 9 yrs on cART). Group 3 - Pre-cART and after treatment interruption.

b 4 Sequences only.

c 3 Sequences only.

d nt/day multiplied by 1000.

**Bold** - significant p value.

### Effect of cART on HIV-1 phylogenetic structure

To further determine if the population shifts detected in the plasma of some patients during and after long-term cART were the result of on-going cycles of virus replication or were due to a shift in the population of cells that express virus particles during therapy, we performed phylogenetic analyses and tests for molecular evolution. Such tests can detect with high sensitivity the emergence of new viral variants indicative of full cycles of replication during cART. We used neighbor-joining trees to first evaluate the direct relationship of the sequences obtained prior to, during, and after therapy and we subsequently used tests for molecular evolution and calculations of root-to-tip distances to detect the emergence of new virus populations during cART.

**Figure 4:**
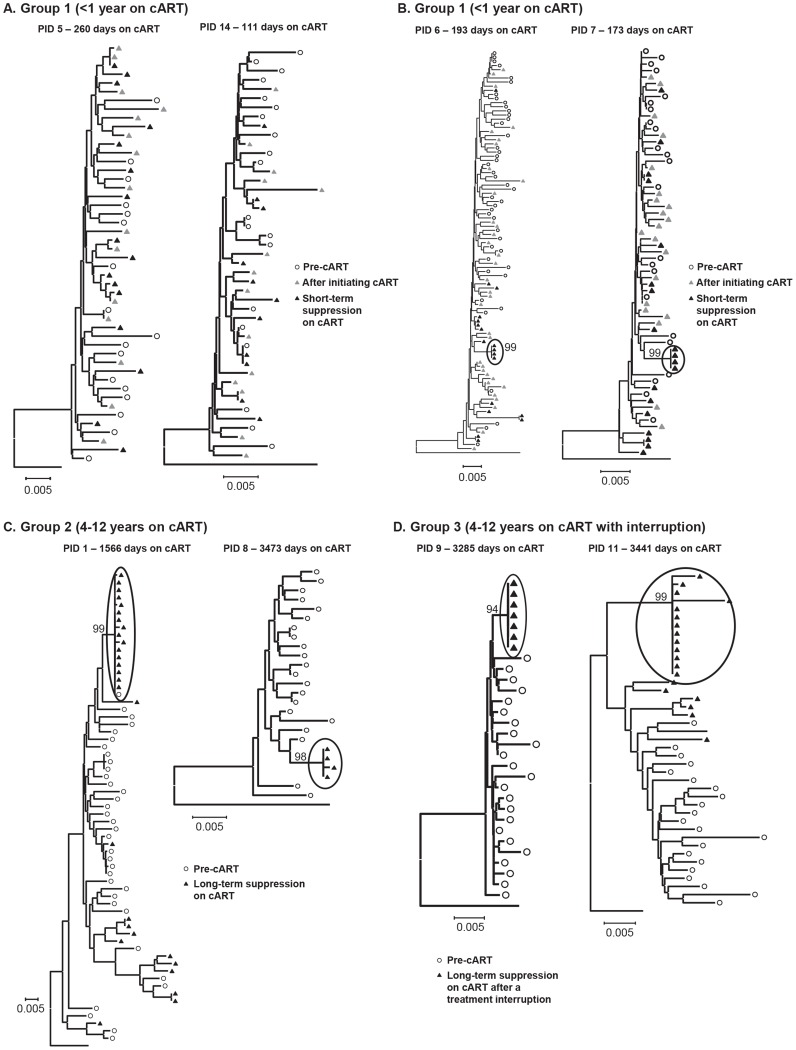
Neighbor-joining trees of single-genome plasma sequences from select samples from (A) Group 1 - short-term cART with no significant divergence of virus population from pre-therapy virus (B) Group 1 - short-term cART with significant divergence of virus during suppression on cART (C) Group 2 – long-term cART and (D) Group 3 – long-term cART, resuppression after a brief treatment interruption. Phylogenetic analyses reveal populations of identical plasma sequences after long-term cART suggesting virus release from a long-lived proliferating cell population.

Figure 4a shows two examples of the population structure in patients in group 1 who had no detectable shift in the virus population using the test for panmixia or the divergence analysis. Consistent with the panmixia results, the structure of sequences obtained during viral decline (gray triangles) and early suppression on cART (black triangles) showed no change from pre-therapy virus (open circles). Figure 4b shows the neighbor-joining trees for the two additional patients in group 1 (PID 6, 7) whose virus had a detectable shift in the population during short-term treatment with cART using the test for panmixia. It is evident from the trees that the shift in population and significant panmixia resulted from clusters of identical sequences that were revealed when levels of viremia were <50 copies/ml (circled black triangles). To confirm that the identical sequences found in PID 6 and 7 resulted in the population shift measured by the test for panmixia, we collapsed the alignment to include only one of each identical sequence and repeated the test. The collapsed alignments resulted in p values of 0.044 and 0.011, respectively, for panmixia (not significant), rejecting the null hypothesis. The revealing of populations of identical sequences during therapy suggests that either a single infected cell is proliferating and releasing virus resulting in a dominant variant appearing in the plasma or that a single variant is expanding through full cycles of replication despite cART. Additional analyses to investigate this question are presented later.

Phylogenetic trees of virus sequences from 6 patients on long-term cART are shown in Figure 4c–d. Trees from representative patients in group 2 (long-term suppression - Figure 4c) and group 3 (re-suppression after brief treatment interruption - Figure 4d) show that the population shift detected by the test for panmixia in these groups resulted from clusters of identical sequences in the plasma (black triangles), and not from additional accumulation of mutations. As noted above, patient 8 was the only one who also showed a significant change in the diversity of the virus population during therapy. The phylogenetic analysis shows that the loss of diversity of the virus population in this patient also resulted from over-representation of identical sequences in the plasma, possibly masking the presence of other viral variants. The presence of identical sequences after long-term cART suggests a proliferating infected cell population as a major source of persistent viremia during therapy. These data also suggest that the virus-producing reservoir of HIV-1 infection may contract during prolonged cART. In addition to the identical sequences, there were also some unique sequences detected in patients in groups 2 and 3 after long-term suppression. The presence of unique variants in the plasma during long-term treatment in PID 1 (Figure 4c) may indicate that on-going replication is another source of residual viremia during therapy in this patient. However, unique variants present in PID 11 (Figure 4c) are more likely due to replication that occurred during the brief treatment interruption in this patient.

**Figure 5:**
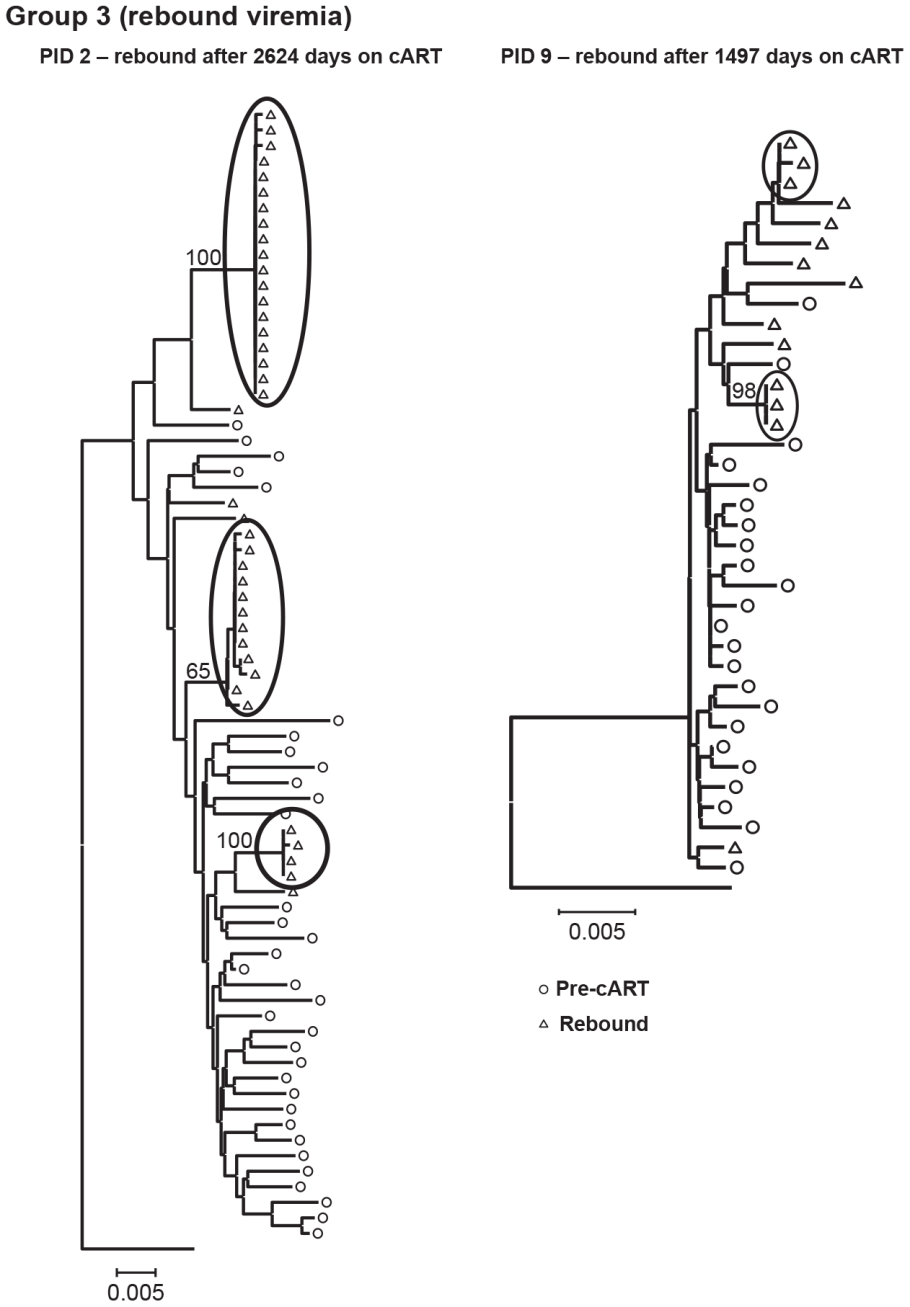
Neighbor-joining trees of single-genome plasma sequences from rebound viremia in patients in Group 3. Phylogenetic analyses reveal populations of identical plasma sequences in rebound viremia after interrupting long-term cART.

The genetics of rebound viremia are shown in two patients from group 3 (PID 2, 9) in Figure 5; and demonstrate that rebounding virus is primarily due to populations of identical sequences, as seen during cART, suggesting a stable, non-evolving reservoir as a likely source of rebound viremia. The presence of multiple populations of rebounding virus argues against the identical sequences persisting during suppression being the source of viral rebound since, in most patients, we detected only a single population of identical variants during suppression. Rebound viremia in these two patients also includes unique variants, some of which may be recombinants between the rebounding rakes of identical sequences and accumulation of new mutations that occurred after interrupting cART.

**Figure 6:**
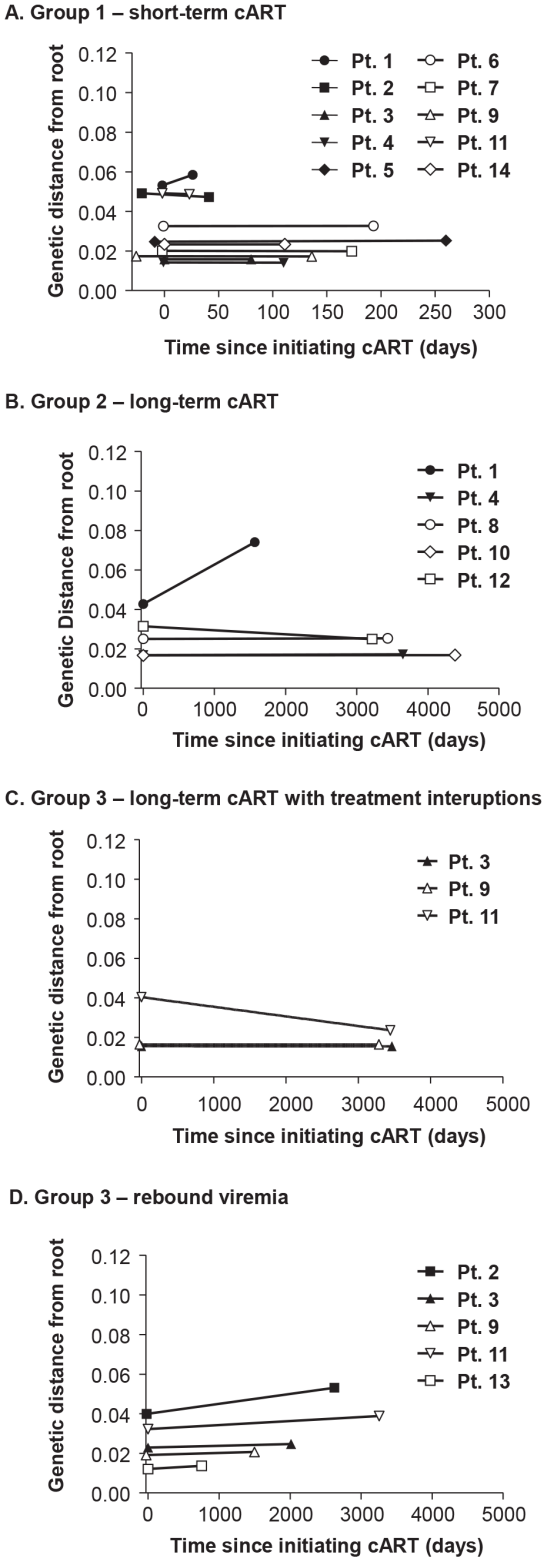
Evolutionary distances of each single-genome sequence from pre-cART and during and after cART compared to the consensus subtype B HIV-1 sequence and plotted over (A) short-term cART (B) long-term cART (C) long-term cART with brief treatment interruptions and (D) rebound viremia. Positive slopes indicate the emergence of new variants and on-going replication during cART. Only one patient (PID 1) showed a positive slope and evidence of molecular evolution during cART.

Neighbor-joining analyses allowed us to visualize the plasma virus populations present during cART compared to those in pretherapy, but cannot be used to determine if the variants present during treatment are newly emergent resulting from full cycles of replication or if they are merely the expression of variants from cells infected prior to treatment. For this purpose, we applied a test for molecular evolution using Bayesian analysis as implemented in BEAST (http://beast.bio.ed.ac.uk) to determine if the plasma virus populations present during therapy were newly emergent variants or were pre-existing. The molecular evolution test was performed by measuring the distances from the root of the tree (rooted on consensus B) to the tip of each branch (Figure 6a–d). If the population structure results from the emergence of new variants, then those sequences will be on branches that are more distant from the root of the tree than variants present in pre-therapy, resulting in positive slopes in Figure 6 as shown in Table 3. The molecular evolution test revealed slopes that were close to 0 (median = 1×10^−5^±4.5×10^−5^ nt/day) with no significant differences between groups (t-test between groups 1 and 2 had p value = 0.72, between groups 2 and 3 p = 0.67, and between groups 1 and 3 p = 0.74), showing that the variants present during the second and third phases of decay and after prolonged therapy were not more distant from the root of the tree than variants present prior to initiating therapy. In a few cases the sequences were actually slightly closer to the root (consensus B) resulting in a negative slope. By contrast, the slopes in untreated elite controllers with similar levels of viremia have significantly positive slopes (median = 15 nt/day) (p = 0.009) when measured over similar intervals [28]. These findings indicate that the viruses with identical sequences that are revealed during cART are not the result of full cycles of replication, but are likely being released from a proliferating cell population that was infected prior to therapy. Although all patients had root-to-tip slopes close to 0, one had a slightly but significantly positive slope after long-term treatment (PID 1, Figure 6a,b, Table 3) suggesting that there is a subset of patients for whom treatment (for some period) is not fully suppressive. The remaining 13/14 patients had slopes not significantly different from zero consistent with complete suppression of viral replication.

## Discussion

The source(s) of persistent viremia during suppressive antiretroviral therapy remains uncertain, and there have been a number of studies to investigate whether repeated full cycles of virus replication occur during adherence to cART or if low-level viremia present in the plasma of successfully treated patients is the result of viral expression from long-lived cells infected prior to treatment. Population genetics and phylogenetic approaches represent powerful techniques to detect genetic change in temporally spaced samples, but in the setting of relatively high genetic diversity it is often difficult to determine whether observed change represents molecular evolution from ongoing replication or a shift in the population of reservoir cells producing virus. One way to resolve this issue is to compare HIV-1 populations prior to and following initiation of cART and to compare temporal changes in viral populations in treated patients to untreated elite controllers with similar levels of viremia and duration of control. In this study, we investigated HIV-1 *gag-pro-pol* populations in infected individuals before, during, and after cART by analyzing the effect of cART on viral genetic diversity and population structure and compared the results to similar data set from a cohort of elite controllers [28]. We previously showed that viral replication and molecular evolution occur in spontaneous HIV-1 elite controllers at levels that are not significantly different from non-controllers [28]. This finding demonstrates that our analytical methods are sensitive enough to detect the emergence of new viral variants despite very low levels of viremia. In fact, with these methods, we are able detect the emergence of new variants even if evolutionary rates are only 10% of those measured in elite controllers (15 nt changes/day). To address the question of ongoing replication during cART, we applied the same analytical approach used in the elite controller cohort [28] to HIV-1 populations in non-controllers on cART for evidence of molecular evolution during treatment. In contrast to our findings in elite controllers, we found clear evidence for virus molecular evolution in only one patient on long-term cART (without treatment interruptions) while we found no evidence for the appearance of new variants in any of the other suppressed patients.

First, we investigated virus populations in samples collected within the first 6 months of initiating cART and compared these populations to the viral sequences obtained from pretherapy samples. We found no change in the diversity, divergence, or phylogenetic structure in populations obtained before and after 6 months of ART, and no evidence of any genetic bottleneck in 8/9 patients on study. These results indicate that both short- and long-lived cellular compartments are seeded with the same viruses and that these compartments are sufficiently large to support highly diverse populations of HIV-1 genomes. The sustained diversity of HIV-1 populations over months of suppressive therapy without a genetic bottleneck or loss of low frequency alleles also implies that pre-existing low-level drug resistance mutations are not likely to be lost during antiretroviral therapy.

To investigate the genetics of persistent HIV-1 during long-term cART, we also sequenced plasma virus populations during 4–15 years of suppressive therapy; and again, compared these populations to those obtained from pretherapy samples. In contrast to earlier samples on cART, we found clusters of identical sequences in plasma samples collected after long-term treatment. However, using phylogenetic tests for molecular evolution (root-to-tip distance analyses), we found no evidence for the appearance of new variants during long-term cART and the clusters fit within the phylogenies of virus populations present before therapy (with the exception of one patient). The presence of identical sequences during ART suggests that virus particles are being produced by an HIV-1 infected clonal cell population, such as stem cell-like CD4+ memory T-cells or other proliferating cell types. These conclusions are consistent with previous studies that indicate that persistent plasma viremia during cART is derived from viral expression in long-lived cells [9], [11] [1] [6], [7], [8]. The specific cell populations giving rise to plasma viremia during cART have not yet been determined but one study nicely demonstrated significantly different populations structures between residual viremia and resting CD4+ cells in 11/13 patients [40] suggesting alternative sources for persistent viremia.

Given that long term therapy reduces the average level of viremia from about 30,000 to about 1–3 copies of RNA per ml on average [1] and that a minority (up to about 1/3) of the sequences in patients on long term therapy are clonal, we can estimate that the cells that produce such virus represent about 1 in 100,000 of the total virus-producing cell population in an untreated individual. Our findings also suggest that these cells are neither expanded nor depleted during therapy as a result of CTL selection. Our observations and those of others (14) that rebound viremia after long-term cART contains homogeneous populations suggests that rebound viremia results from the expansion of identical sequences present during suppression or from a small number of founder viruses (as seen in acute infection). Further experiments are required to determine the relationship of virus populations that persist during therapy to those that rebound after treatment interruption.

The conclusion that cART effectively and completely halts HIV-1 replication in those infected cells that are responsible for viremia is consistent with prior studies by us and others showing that low levels of viremia on therapy are independent of the therapeutic regimen used and they cannot be further suppressed by additional drugs [6], [7], [8]. Our conclusions are also consistent with the initial observations of Persaud and coworkers who demonstrated that drug resistant mutations do not emerge in patients with suppressed viremia [41]. Several observations, including transient increases in 2LTR circles in some cART treated patients undergoing raltegravir intensification, and studies measuring relative levels of HIV-1 RNA in specific compartments [42] have suggested the presence of localized, limited HIV-1 replication. However, the relationship between the 2-LTR circles and low level viremia has not been firmly established. It is likely that a very small fraction of the virions released during suppressive cART give rise to the 2 LTR circles and that these represent dead-end events, not continuous replication, most likely related to the use of a specific antiviral treatment regimen [42]. Our findings here suggest that low level viremia persisting during cART results primarily from expression of virus in expanding cell populations infected prior to initiating therapy. Cure of HIV-1 infection will require strategies that either eliminate the extremely rare cell population that can chronically produce infectious virus or prevent regrowth of virus from these reservoirs following cessation of cART.

## Supporting Information

Table S1 Description of patient samples analyzed.
(DOCX)

Table S2 Patient HLA types and shifts in CTL Mutations during cART.
(DOCX)

## References

[1] Palmer S, Maldarelli F, Wiegand A, Bernstein B, Hanna GJ, et al (2008) Low-level viremia persists for at least 7 years in patients on suppressive antiretroviral therapy. Proc Natl Acad Sci U S A 105: 3879–3884. 18332425 10.1073/pnas.0800050105 PMC2268833

[2] Maldarelli F, Palmer S, King MS, Wiegand A, Polis MA, et al (2007) ART suppresses plasma HIV-1 RNA to a stable set point predicted by pretherapy viremia. PLoS Pathog 3: e46. 17411338 10.1371/journal.ppat.0030046 PMC1847689

[3] Coffin JM (1995) HIV population dynamics in vivo: implications for genetic variation, pathogenesis, and therapy. Science 267: 483–489. 7824947 10.1126/science.7824947

[4] Coffin JM (1996) HIV viral dynamics. Aids 10 Suppl 3: S75–84. 8970715

[5] Perelson AS, Essunger P, Cao Y, Vesanen M, Hurley A, et al (1997) Decay characteristics of HIV-1-infected compartments during combination therapy. Nature 387: 188–191. 9144290 10.1038/387188a0

[6] Dinoso JB, Kim SY, Wiegand AM, Palmer SE, Gange SJ, et al (2009) Treatment intensification does not reduce residual HIV-1 viremia in patients on highly active antiretroviral therapy. Proc Natl Acad Sci U S A 106: 9403–9408. 19470482 10.1073/pnas.0903107106 PMC2685743

[7] Gandhi RT, Zheng L, Bosch RJ, Chan ES, Margolis DM, et al (2010) The effect of raltegravir intensification on low-level residual viremia in HIV-infected patients on antiretroviral therapy: a randomized controlled trial. PLoS Med 7 8: e1000321. 20711481 10.1371/journal.pmed.1000321 PMC2919424

[8] McMahon D, Jones J, Wiegand A, Gange SJ, Kearney M, et al (2010) Short-course raltegravir intensification does not reduce persistent low-level viremia in patients with HIV-1 suppression during receipt of combination antiretroviral therapy. Clin Infect Dis 50: 912–919. 20156060 10.1086/650749 PMC2897152

[9] Bailey JR, Sedaghat AR, Kieffer T, Brennan T, Lee PK, et al (2006) Residual human immunodeficiency virus type 1 viremia in some patients on antiretroviral therapy is dominated by a small number of invariant clones rarely found in circulating CD4+ T cells. J Virol 80: 6441–6457. 16775332 10.1128/JVI.00591-06 PMC1488985

[10] Wagner TA, McKernan JL, Tobin NH, Tapia KA, Mullins JI, et al (2013) An increasing proportion of monotypic HIV-1 DNA sequences during antiretroviral treatment suggests proliferation of HIV-infected cells. Journal of Virology 87: 1770–1778. 23175380 10.1128/JVI.01985-12 PMC3554159

[11] Joos B, Fischer M, Kuster H, Pillai SK, Wong JK, et al (2008) HIV rebounds from latently infected cells, rather than from continuing low-level replication. Proceedings of the National Academy of Sciences of the United States of America 105: 16725–16730. 18936487 10.1073/pnas.0804192105 PMC2575487

[12] Shiu C, Cunningham CK, Greenough T, Muresan P, Sanchez-Merino V, et al (2009) Identification of Ongoing HIV-1 Replication in Residual Viremia during Recombinant HIV-1 Poxvirus Immunizations in Patients with Clinically Undetectable Viral Loads on Durable Suppressive HAART. J Virol 83: 9731–42. 19605490 10.1128/JVI.00570-09 PMC2748010

[13] Chun TW, Nickle DC, Justement JS, Meyers JH, Roby G, et al (2008) Persistence of HIV in gut-associated lymphoid tissue despite long-term antiretroviral therapy. J Infect Dis 197: 714–720. 18260759 10.1086/527324

[14] Gunthard HF, Wong JK, Ignacio CC, Guatelli JC, Riggs NL, et al (1998) Human immunodeficiency virus replication and genotypic resistance in blood and lymph nodes after a year of potent antiretroviral therapy. J Virol 72: 2422–2428. 9499103 10.1128/jvi.72.3.2422-2428.1998 PMC109542

[15] Benito JM, Lopez M, Lozano S, Martinez P, Gonzalez-Lahoz J, et al (2004) CD38 expression on CD8 T lymphocytes as a marker of residual virus replication in chronically HIV-infected patients receiving antiretroviral therapy. AIDS Res Hum Retroviruses 20: 227–233. 15018711 10.1089/088922204773004950

[16] Cohen Stuart JW, Hazebergh MD, Hamann D, Otto SA, Borleffs JC, et al (2000) The dominant source of CD4+ and CD8+ T-cell activation in HIV infection is antigenic stimulation. J Acquir Immune Defic Syndr 25: 203–211. 11115950 10.1097/00126334-200011010-00001

[17] Martinez E, Arnedo M, Giner V, Gil C, Caballero M, et al (2001) Lymphoid tissue viral burden and duration of viral suppression in plasma. Aids 15: 1477–1482. 11504979 10.1097/00002030-200108170-00004

[18] Ruiz L, van Lunzen J, Arno A, Stellbrink HJ, Schneider C, et al (1999) Protease inhibitor-containing regimens compared with nucleoside analogues alone in the suppression of persistent HIV-1 replication in lymphoid tissue. Aids 13: F1–8. 10207538 10.1097/00002030-199901140-00001

[19] Martinez MA, Cabana M, Ibanez A, Clotet B, Arno A, et al (1999) Human immunodeficiency virus type 1 genetic evolution in patients with prolonged suppression of plasma viremia. Virology 256: 180–187. 10191182 10.1006/viro.1999.9601

[20] Llewellyn N, Zioni R, Zhu H, Andrus T, Xu Y, et al (2006) Continued evolution of HIV-1 circulating in blood monocytes with antiretroviral therapy: genetic analysis of HIV-1 in monocytes and CD4+ T cells of patients with discontinued therapy. J Leukoc Biol 80: 1118–1126. 17056765 10.1189/jlb.0306144

[21] Shi B, Kitchen C, Weiser B, Mayers D, Foley B, et al Evolution and recombination of genes encoding HIV-1 drug resistance and tropism during antiretroviral therapy. Virology 404: 5–20. 20451945 10.1016/j.virol.2010.04.008 PMC3186207

[22] Chun TW, Davey RT Jr, Ostrowski M, Shawn Justement J, Engel D, et al (2000) Relationship between pre-existing viral reservoirs and the re-emergence of plasma viremia after discontinuation of highly active anti-retroviral therapy. Nat Med 6: 757–761. 10888923 10.1038/77481

[23] Buzon MJ, Massanella M, Llibre JM, Esteve A, Dahl V, et al (2010) HIV-1 replication and immune dynamics are affected by raltegravir intensification of HAART-suppressed subjects. Nature Medicine 16: 460–465. 10.1038/nm.2111 20228817

[24] Hatano H, Hayes TL, Dahl V, Sinclair E, Lee TH, et al (2011) A randomized, controlled trial of raltegravir intensification in antiretroviral-treated, HIV-infected patients with a suboptimal CD4+ T cell response. The Journal of infectious diseases 203: 960–968. 21402547 10.1093/infdis/jiq138 PMC3068029

[25] Llibre JM, Buzon MJ, Massanella M, Esteve A, Dahl V, et al (2012) Treatment intensification with raltegravir in subjects with sustained HIV-1 viraemia suppression: a randomized 48-week study. Antiviral therapy 17: 355–364. 22290239 10.3851/IMP1917

[26] Polis MA, Sidorov IA, Yoder C, Jankelevich S, Metcalf J, et al (2001) Correlation between reduction in plasma HIV-1 RNA concentration 1 week after start of antiretroviral treatment and longer-term efficacy. Lancet 358: 1760–1765. 11734232 10.1016/s0140-6736(01)06802-7

[27] Maldarelli F, Kearney M, Palmer S, Stephens R, Mican J, et al (2013) HIV Populations are Large and Accumulate High Genetic Diversity in Nonlinear Fashion. Journal of Virology 87 18: 10313–23. 23678164 10.1128/JVI.01225-12 PMC3754011

[28] Mens H, Kearney M, Wiegand A, Shao W, Schonning K, et al (2010) HIV-1 continues to replicate and evolve in patients with natural control of HIV infection. Journal of Virology 84: 12971–12981. 20926564 10.1128/JVI.00387-10 PMC3004307

[29] Elbeik T, Alvord WG, Trichavaroj R, de Souza M, Dewar R, et al (2002) Comparative analysis of HIV-1 viral load assays on subtype quantification: Bayer Versant HIV-1 RNA 3.0 versus Roche Amplicor HIV-1 Monitor version 1.5. J Acquir Immune Defic Syndr 29: 330–339. 11917236 10.1097/00126334-200204010-00002

[30] Palmer S, Kearney M, Maldarelli F, Halvas EK, Bixby CJ, et al (2005) Multiple, linked human immunodeficiency virus type 1 drug resistance mutations in treatment-experienced patients are missed by standard genotype analysis. J Clin Microbiol 43: 406–413. 15635002 10.1128/JCM.43.1.406-413.2005 PMC540111

[31] Kearney M, Palmer S, Maldarelli F, Shao W, Polis MA, et al (2008) Frequent Polymorphism at Drug Resistance Sites in HIV-1 Protease and Reverse Transcriptase. AIDS 22 4: 497–501. 18301062 10.1097/QAD.0b013e3282f29478 PMC2921824

[32] Kearney M, Maldarelli F, Shao W, Margolick JB, Daar ES, et al (2009) Human immunodeficiency virus type 1 population genetics and adaptation in newly infected individuals. J Virol 83: 2715–2727. 19116249 10.1128/JVI.01960-08 PMC2648286

[33] Tamura K, Peterson D, Peterson N, Stecher G, Nei M, et al (2011) MEGA5: molecular evolutionary genetics analysis using maximum likelihood, evolutionary distance, and maximum parsimony methods. Molecular Biology and Evolution 28: 2731–2739. 21546353 10.1093/molbev/msr121 PMC3203626

[34] Achaz G, Palmer S, Kearney M, Maldarelli F, Mellors JW, et al (2004) A robust measure of HIV-1 population turnover within chronically infected individuals. Mol Biol Evol 21: 1902–1912. 15215321 10.1093/molbev/msh196

[35] Rouzine IM, Coffin JM (2010) Multi-site adaptation in the presence of infrequent recombination. Theoretical Population Biology 77: 189–204. 20149814 10.1016/j.tpb.2010.02.001 PMC2849900

[36] Hudson RR, Boos DD, Kaplan NL (1992) A statistical test for detecting geographic subdivision. Mol Biol Evol 9: 138–151. 1552836 10.1093/oxfordjournals.molbev.a040703

[37] Swofford DL (2003) PAUP: Phylogenetic analysis using parsimony, version 4. Sunderland (Massachusetts): Sinauer.

[38] Cornuet JM, Luikart G (1996) Description and power analysis of two tests for detecting recent population bottlenec.ks from allele frequency data. Genetics 144: 2001–2014. 8978083 10.1093/genetics/144.4.2001 PMC1207747

[39] Zhang Q, Wang P, Kim Y, Haste-Andersen P, Beaver J, et al (2008) Immune epitope database analysis resource (IEDB-AR). Nucleic Acids Research 36: W513–518. 18515843 10.1093/nar/gkn254 PMC2447801

[40] Brennan TP, Woods JO, Sedaghat AR, Siliciano JD, Siliciano RF, et al (2009) Analysis of human immunodeficiency virus type 1 viremia and provirus in resting CD4+ T cells reveals a novel source of residual viremia in patients on antiretroviral therapy. Journal of Virology 83: 8470–8481. 19535437 10.1128/JVI.02568-08 PMC2738142

[41] Nettles RE, Kieffer TL, Kwon P, Monie D, Han Y, et al (2005) Intermittent HIV-1 viremia (Blips) and drug resistance in patients receiving HAART. JAMA : the journal of the American Medical Association 293: 817–829. 15713771 10.1001/jama.293.7.817

[42] Yukl SA, Gianella S, Sinclair E, Epling L, Li Q, et al (2010) Differences in HIV burden and immune activation within the gut of HIV-positive patients receiving suppressive antiretroviral therapy. The Journal of infectious diseases 202: 1553–1561. 20939732 10.1086/656722 PMC2997806

