# Supplementary material for: Lack of Detectable HIV-1 Molecular Evolution during Suppressive Antiretroviral Therapy
Source: PLoS Pathog. 2014 Mar 20;10(3):e1004010. doi: 10.1371/journal.ppat.1004010 (PMC3961343; doi:10.1371/journal.ppat.1004010)
Supplement: Table S1 — Description of patient samples analyzed. (DOCX) [file ppat.1004010.s001.docx]

**Table S1. Patient Samples**

| **PID** | **Group^a^** | **Sample Date** | **HIV RNA Copies/ml** | **Time Since Starting Therapy (Days)** | **Single-Genome Sequences (Number)** |
| --- | --- | --- | --- | --- | --- |
| 1 | 1, 2 | 12/11/02 | 163,006 | -2 | 25 |
|  |  | 12/13/02 | 31,591 | 0 | 36 |
|  |  | 12/18/02 | 2,767 | 5 | 18 |
|  |  | 12/30/02 | 358 | 7 | 15 |
|  |  | 01/08/03 | 101 | 26 | 25 |
|  |  | 03/26/03 | <50 | 103 | 2 |
|  |  | 04/30/03 | <50 | 138 | 4 |
|  |  | 05/28/03 | <50 | 166 | 2 |
|  |  | 06/26/03 | <50 | 195 | 4 |
|  |  | 03/29/07 | <50 | 1566 | 27 |
|  |  | 04/11/08 | <50 | 1946 | 4 |
| 2 | 1, 3 | 07/10/02 | 217959 | -21 | 24 |
|  |  | 08/01/02 | 218539 | 1 | 34 |
|  |  | 08/06/02 | 3833 | 6 | 17 |
|  |  | 09/10/02 | 137 | 41 | 9 |
|  |  | 10/01/02 | 62 | 63 | 3 |
|  |  | 10/07/09 | 828 | 2624 | 39 |
| 3 | 1, 3 | 01/27/00 | 48682 | 0 | 16 |
|  |  | 04/17/00 | 308 | 80 | 7 |
|  |  | 11/01/05 | 38967 | 2013 | 23 |
|  |  | 07/23/09 | <50 | 3465 | 4 |
| 4 | 1, 2 | 02/02/99 | 21836 | -1 | 21 |
|  |  | 02/04/99 | 69132 | 1 | 28 |
|  |  | 02/18/99 | 723 | 14 | 12 |
|  |  | 03/02/99 | 472 | 26 | 19 |
|  |  | 03/17/99 | 499 | 41 | 19 |
|  |  | 05/25/99 | <50 | 110 | 7 |
|  |  | 06/22/99 | <50 | 138 | 2 |
|  |  | 07/20/99 | <50 | 166 | 6 |
|  |  | 10/06/09 | <50 | 3650 | 24 |
| 5 | 1 | 10/16/00 | 1938350 | -9 | 10 |
|  |  | 10/17/00 | 1391370 | -8 | 8 |
|  |  | 10/19/00 | 2808580 | -6 | 6 |
|  |  | 10/25/00 | 1801380 | 0 | 11 |
|  |  | 10/31/00 | 71560 | 6 | 19 |
|  |  | 11/15/00 | 20425 | 22 | 26 |
|  |  | 11/22/00 | 15157 | 29 | 15 |
|  |  | 03/02/01 | 136 | 111 | 8 |
|  |  | 03/16/01 | 126 | 125 | 5 |
|  |  | 04/13/01 | 62 | 170 | 7 |
|  |  | 05/11/01 | <50 | 198 | 4 |
|  |  | 07/13/01 | <50 | 260 | 12 |
|  |  | 08/30/02 | <50 | 409 | 4 |
| 6 | 1 | 01/26/99 | 329276 | -1 | 45 |
|  |  | 02/02/99 | 7334 | 6 | 34 |
|  |  | 02/11/99 | 839 | 15 | 7 |
|  |  | 02/23/99 | 161 | 27 | 23 |
|  |  | 06/14/99 | <50 | 138 | 18 |
|  |  | 08/09/99 | <50 | 193 | 9 |
| 7 | 1 | 07/13/99 | 500,000 | -2 | 23 |
|  |  | 07/18/99 | 129398 | 4 | 18 |
|  |  | 07/23/99 | 15377 | 9 | 13 |
|  |  | 08/03/99 | 4255 | 20 | 21 |
|  |  | 08/06/99 | 2698 | 23 | 2 |
|  |  | 08/10/99 | 745 | 27 | 24 |
|  |  | 09/14/99 | 128 | 62 | 25 |
|  |  | 10/04/99 | <50 | 82 | 3 |
|  |  | 12/02/99 | 59 | 140 | 12 |
|  |  | 01/04/00 | <50 | 173 | 11 |
|  |  | 01/04/00 | <50 | 173 | 8 |
| 8 | 2 | 04/17/00 | 4504 | -3 | 21 |
|  |  | 09/14/09 | <50 | 3437 | 4 |
| 9 | 1, 3 | 09/22/00 | 39936 | -26 | 32 |
|  |  | 10/19/00 | 78675 | 1 | 23 |
|  |  | 10/24/00 | 3490 | 6 | 34 |
|  |  | 11/02/00 | 678 | 15 | 21 |
|  |  | 11/09/00 | 578 | 22 | 7 |
|  |  | 03/05/01 | <50 | 136 | 16 |
|  |  | 04/11/05 | 58439 | 1497 | 14 |
|  |  | 08/17/09 | <50 | 3285 | 7 |
| 10 | 2 | 12/09/97 | 87280 | -1 | 17 |
|  |  | 08/20/09 | <50 | 4380 | 22 |
| 11 | 1, 3 | 07/11/00 | 289173 | -2 | 22 |
|  |  | 07/13/00 | 385687 | 1 | 40 |
|  |  | 07/18/00 | 13026 | 6 | 12 |
|  |  | 07/27/00 | 2206 | 15 | 3 |
|  |  | 08/04/00 | 751 | 23 | 19 |
|  |  | 06/11/09 | 15521 | 3255 | 21 |
|  |  | 12/14/09 | <50 | 3441 | 19 |
| 12 | 2 | 07/08/97 | 12990 | 1 | 14 |
|  |  | 08/27/99 | <50 | 780 | 3 |
|  |  | 10/05/09 | <50 | 3215 | 3 |
| 13 | 3 | 06/30/98 | 19280 | -1 | 24 |
|  |  | 05/30/00 | <50 | 700 |  |
|  |  | 07/25/00 | 373 | 755 | 23 |
| 14 | 1 | 09/15/01 | 499326 | 0 | 20 |
|  |  | 10/26/01 | 238 | 41 | 14 |
|  |  | 11/09/01 | 89 | 54 | 11 |
|  |  | 12/07/01 | <50 | 82 | 2 |
|  |  | 01/04/02 | <50 | 111 | 10 |
| **^a^** Group definitions:  Group 1 - samples analyzed pre-cART and <1 yr on cART  Group 2 - pre-cART and after long-term suppression on cART (average 9 yrs on cART)  Group 3 - pre-cART and after treatment interruption | | | | | |
