# Supplementary material for: Lack of Detectable HIV-1 Molecular Evolution during Suppressive Antiretroviral Therapy
Source: PLoS Pathog. 2014 Mar 20;10(3):e1004010. doi: 10.1371/journal.ppat.1004010 (PMC3961343; doi:10.1371/journal.ppat.1004010)
Supplement: Table S2 — Patient HLA types and shifts in CTL Mutations during cART. (DOCX) [file ppat.1004010.s002.docx]

**Table S2. Shifts in CTL Mutations during cART**

|  |  |  |  |  | **Positions with Significant Change in Frequency between Pretherapy and Suppression** | | |  |  |
| --- | --- | --- | --- | --- | --- | --- | --- | --- | --- |
| **PID** | **Time between Samples (Days)** | **HLA-A** | **HLA-B** | **Polymorphic Loci (Number)** | **Total (Number)** |  | **Percent** | **Shift to a Sensitive CTL Site Sequence** | **Shift to Escape CTL Site Sequence** |
| **14** | 111 | A02, A24 | B35.01, B49.01 | 36 | 1 |  | 2.8 | 0 | 0 |
| **9** | 136 | A02, A68 | B40, B44 | 32 | 0 |  | <3 |  |  |
| **7** | 173 | A03, A24 | B35 | 29 | 0 |  | <3 |  |  |
| **6** | 193 | A30 | B35, B42 | 44 | 3 |  | 6.8 | 2 | 3 |
| **5** | 409 | A116, A601 | B15, B58 | 68 | 0 |  | <1.5 |  |  |
| **1** | 1946 | A11 | B44/B07 | 36 | 4 |  | 11.1 | 0 | 0 |
| **4** | 3650 | 8001* | B18/B8 | 33 | 2 |  | 6.1 | 1 | 1 |
| **10** | 4380 | A01, A02 | B07, B08 | 69 | 0 |  | <1.5 |  |  |
| ^a^Analysis includes only patients who had 7 or more sequences at two distant time points during suppression without treatment interruptions  *CTL epitopes not available | | | | | | | | | |
